# Interaction between Lysophosphatidic Acid, Prostaglandins and the Endocannabinoid System during the Window of Implantation in the Rat Uterus

**DOI:** 10.1371/journal.pone.0046059

**Published:** 2012-09-28

**Authors:** Micaela S. Sordelli, Jimena S. Beltrame, Maximiliano Cella, María Gracia Gervasi, Silvina Perez Martinez, Juliana Burdet, Elsa Zotta, Ana M. Franchi, María Laura Ribeiro

**Affiliations:** 1 Laboratorio de Fisiología y Farmacología de la Reproducción, CEFYBO (CONICET – Facultad de Medicina, Universidad de Buenos Aires (UBA)), Buenos Aires, Argentina; 2 Laboratorio de Fisiopatología de la Preñez y el Parto, CEFYBO (CONICET – Facultad de Medicina, UBA), Buenos Aires, Argentina; 3 Laboratorio de Biología de la Reproducción en Mamíferos, CEFYBO (CONICET – Facultad de Medicina, UBA), Buenos Aires, Argentina; 4 Laboratorio de Fisiopatología Molecular (Cátedra de Fisiopatología, Departamento de Cs. Biológicas, Facultad de Farmacia y Bioquıímica, UBA), Buenos Aires, Argentina; French National Centre for Scientific Research, France

## Abstract

Bioactive lipid molecules as lysophosphatidic acid (LPA), prostaglandins (PG) and endocannabinoids are important mediators of embryo implantation. Based on previous published data we became interested in studying the interaction between these three groups of lipid derivatives in the rat uterus during the window of implantation. Thus, we adopted a pharmacological approach *in vitro* using LPA, DGPP (a selective antagonist of LPA3, an LPA receptor), endocannabinoids’ receptor selective antagonists (AM251 and AM630) and non selective (indomethacin) and selective (NS-398) inhibitors of cyclooxygenase-1 and 2 enzymes. Cyclooxygenase isoforms participate in prostaglandins’ synthesis. The incubation of the uterus from rats pregnant on day 5 of gestation (implantation window) with LPA augmented the activity and the expression of fatty acid amide hydrolase, the main enzyme involved in the degradation of endocannabinoids in the rodent uteri, suggesting that LPA decreased endocannabinoids’ levels during embryo implantation. It has been reported that high endocannabinoids are deleterious for implantation. Also, LPA increased PGE2 production and cyclooxygenase-2 expression. The incubation of LPA with indomethacin or NS-398 reversed the increment in PGE2 production, suggesting that cyclooxygenase-2 was the isoform involved in LPA effect. PGs are important mediators of decidualization and vascularization at the implantation sites. All these effects were mediated by LPA3, as the incubation with DGPP completely reversed LPA stimulatory actions. Besides, we also observed that endocannabinoids mediated the stimulatory effect of LPA on cyclooxygenase-2 derived PGE2 production, as the incubation of LPA with AM251 or AM630 completely reversed LPA effect. Also, LPA augmented via LPA3 decidualization and vascularization markers. Overall, the results presented here demonstrate the participation of LPA3 in the process of implantation through the interaction with other groups of lipid molecules, prostaglandins and endocannabinoids, which prepare the uterine milieu for embryo invasion during the window of implantation.

## Introduction

Virtually all aspects of cellular function are regulated by lipids, which are typically derived enzymatically from abundant substrates in the cellular or extracellular environment. Experiments in mice have directly shown that lipid molecules are essential during embryo invasion (for details see review [1]). The quality of implantation determines the quality of pregnancy and fetal well-being and failure to achieve ‘on-time’ implantation risks pregnancy outcome.

**Table 1:** RT-PCR primers and conditions.

mRNA	Primers	Conditions
**β-actina**	Sense 5′-GCCATGTACGTAGCCATCC-3′	94°C, 5 min - 59°C, 30 seg - 72°C, 1 min
	Antisense 5′-CTCTCAGCTGTGGTGGTGAA-3′	
**LPA3**	Sense 5′-TGAGCCTCCATGTGTAGCTG-3′	94°C, 5 min - 59°C, 30 seg - 72°C, 1 min
	Antisense 5′-TTCTACACCTCCACCCTTGC-3′	
**Lyso-PLD**	Sense 5′-AAAAGGGCCGTACTTGTGTC-3′	94°C, 5 min - 57°C, 30 seg - 72°C, 1 min
	Antisense 5′-TGCTTCGTTCCATCTACGAG-3′	
**FAAH**	Sense 5′-GAGGCTGGCTTTCAACTCAC-3′	94°C, 5 min - 57°C, 30 seg - 72°C, 1 min
	Antisense 5′-TCTTCCCTGGTCCAGTGTTC-3′	
**COX-1**	Sense 5′-ATAGAGATGGGGGCTCCTTT-3′	94°C, 5 min - 57°C, 30 seg - 72°C, 1 min
	Antisense 5′-ACACGGAAGGAGACATAGGG-3′	
**COX-2**	Sense 5′-GGCCATGGAGTGGACTTAAA-3′	94°C, 5 min - 59°C, 30 seg - 72°C, 1 min
	Antisense 5′-CTCTCCACCGATGACCTGAT-3′	
**IGFBP-1**	Sense 5′-GCGGTAGTGCCTAGAACGAG-3′	94°C, 5 min - 59°C, 30 seg - 72°C, 1 min
	Antisense 5′-TGGGATTCGATGAGGAAGTC-3′	
**IL-10**	Sense 5′-AAGGACCAGCTGGACAAACAT-3′	94°C, 5 min - 59°C, 30 seg - 72°C, 1 min
	Antisense 5′-AGGGGAGAAATCGATGACAG-3′	

**Table 2:** First and the second antibody dilutions employed in Western Blot analyses.

Protein detected	First antibody	Second antibody
**LPA3**	1∶250	1∶10000
**Lyso-PLD**	1∶200	1∶3000
**FAAH**	1∶150	1∶15000
**COX-1**	1∶250	1∶10000
**COX-2**	1∶250	1∶10000
**β-Actina**	1∶4000	1∶10000

Some of the most widely studied lipid mediators are the phosphorylated lipids such as lysophosphatidic acid (LPA). This ligand has pleiotropic actions in many cells and tissues, exerted through binding to multiple G-protein coupled receptors, as LPA3. Targeted deletion of LPA3 in mice, results in significantly reduced litter size and altered embryo spacing, which could be attributed to delayed implantation and altered embryo spacing [2]. These two events lead to delayed embryonic development, hypertrophic placentas shared by multiple embryos and embryonic death. An enzyme previously demonstrated to influence implantation, cyclooxygenase-2 [3], is downregulated in LPA3-deficient uteri during preimplantation. Two cyclooxygenase (COX) isoforms have been described, COX-1 and COX-2, which are rate limiting in the production of fatty acid derivatives known as prostaglandins (PGs). In LPA3^−/−^ mice, down regulation of COX-2 leads to reduced levels of PGs, which have been shown to be relevant at implantation [3], [4]. COX-2 is restricted to implantation sites in most species studied and COX-2^−/−^ mice have defective implantation and decidualization [3], [5]. PGE2 and PGI2 increase vascular permeability and decidualization at the implantation sites [6], [7] and exogenous administration of PGE2 and PGI2 into LPA3^−/−^ females rescues delayed implantation but did not rescue defects in embryo spacing [2], [8]. Other authors have observed that LPA stimulates the expression of COX-2 mRNA in the porcine endometrium [9] and increases the synthesis of PGE2 in the ovine trophectoderm and in the bovine endometrium [10], [11]. These data identify LPA3 receptor-mediated signaling as a new influence on implantation and further indicate linkage between LPA signaling and PGs biosynthesis. Tokumura and colleagues [12], [13] described that LPA and lysophospholipase-D (Lyso-PLD), the major lysophospholipid generating enzyme, increase in women serum with the progress of gestation. Also, the expression of this enzyme has been localized in human placenta, especially in trophoblast cells [14].

Anandamide (N-arachidonoylethanolamine, AEA) and 2-arachidonoyl glycerol (2-AG) are two endocannabinoid ligands for the cannabinoid receptors type 1 (CB1) and type 2 (CB2) [15], [16]. A physiological tone of AEA and 2-AG are critical to preimplantation events in mice, since either silencing or amplification of these signaling pathways causes retarded development and oviductal retention of embryos via CB1, leading to deferred implantation and compromised pregnancy outcome [5], [17]–[19]. Genetic evidence suggests that fatty acid amide hydrolase (FAAH) is the major degrading enzyme for endocannabinoids [19]. Aberrant functioning of these pathways impacting uterine AEA and/or 2-AG levels or effects would compromise pregnancy outcome. In fact, low FAAH and high AEA levels are associated with failure to achieve an ongoing pregnancy after *in vitro* fertilization and embryo transfer [20]. Recently, we have observed that AEA increases PGE2 and PGF2α production via CB2 receptors in the receptive rat uterus [21].

In order to gain more insight into the contribution of these bioactive lipid mediators to the crucial events leading to implantation, the aim of the present work was to investigate which factors contribute to LPA3 receptor-specific role during the window of implantation. Our results suggest that LPA through binding to LPA3, modulated the levels of important lipid mediators, endocannabinoids and prostaglandins, that prepare the uterine milieu for embryo invasion during the window of implantation.

**Figure 1:**
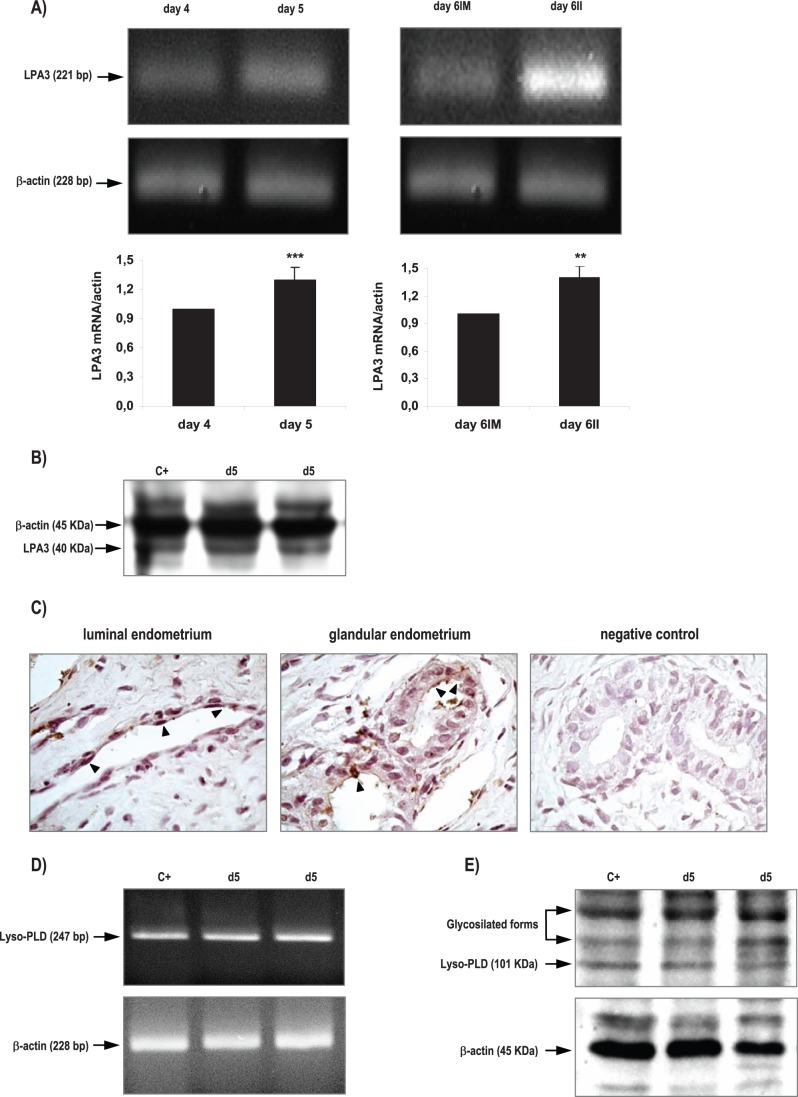
Expression of LPA3 receptor and Lyso-PLD enzyme in the rat uterus during the window of implantation. LPA3 messenger (A) and protein (B) and Lyso-PLD messenger (D) and protein (E) were detected by RT-PCR and Western Blot. Also, LPA3 localization (C) was determined by immunhistochemistry in day 5. Black arrows indicate positive staining (200x). In A *** p<0.001 day 4 vs day 5, ** p<0,01 day 6IM vs day 6II. Results are shown as means ± sem. N = 4–6 for each point. d5: uterus from rats pregnant on day 5 of gestation; IM: implantation sites; II: inter-implantation sites.

## Materials and Methods

### Drugs and Chemicals

Dulbecco’s Modified Eagle Medium (DMEM) without phenol red, fetal calf serum, penicillin G, streptomycin and amphotericin B were purchased to GIBCO (Invitrogen, Buenos Aires, Argentina). RTU Vectastain kit and diaminobenzidine were from Vector (Peterborough, UK). [5], [6], [8], [9], [11], [12], [14], [15](n)-[^3^H]-PGF_2α_ (160 Ci/mmol, 200 µCi/ml) and [5], [6], [8], [9], [11], [12], [14], [15](n)-[^3^H]-PGE_2_ (130 Ci/mmol, 100 µCi/ml) were provided by Amersham Corporation (Migliore Laclaustra, Buenos Aires, Argentina). [^3^H]-anandamide (172.4 Ci/mmol, 100 µCi/ml) and Optiphase-3 scintillation solution were provided by Perkin Elmer (ETC, Buenos Aires, Argentina). TLC aluminum silica gel plates were purchased from Merck KGaA (Darmstadt, Germany). Anandamide, Diacylglycerol pyrophosphate (DGPP 8∶0), PGF2α and PGE2 antiserum, luminol (Fluka), p-coumaric acid (Fluka) and western blot detergents and inhibitors were purchased to Sigma Chemical Company (Buenos Aires, Argentina) and Bio Rad (Tecnolab, Buenos Aires, Argentina). Indomethacin was from Montpellier (Argentina). NS-398, 1-oleoyl-lysophosphatidic acid (18∶0) and the first antibodies against LPA3, Lyso-PLD, COX-1 and COX-2 were from Cayman Chemical (Migliore Laclaustra, Buenos Aires, Argentina). AM251 and AM630 were purchased in Tocris Cookson Inc. (Ellisville, MO, USA). Trizol reagent was from Genbiotech (Buenos Aires, Argentina). RNAse free DNAse I, Moloney Murine Leukemia virus reverse transcriptase (MMLV-RT) and random primers were purchased from Invitrogen (Buenos Aires, Argentina). Goat anti-rabbit horseradish peroxidase-conjugated IgG second antibodies were from Jackson ImmunoResearch Laboratories, Inc. (Sero-Immuno Diagnostics INC, Tucker, GA, USA) and Sigma (Buenos Aires, Argentina). FAAH first antibody was a kind gift from Dr. Benjamin Cravatt (Department of Chemical Physiology, The Scripps Research Institute). All other chemicals were analytical grade.

**Figure 2:**
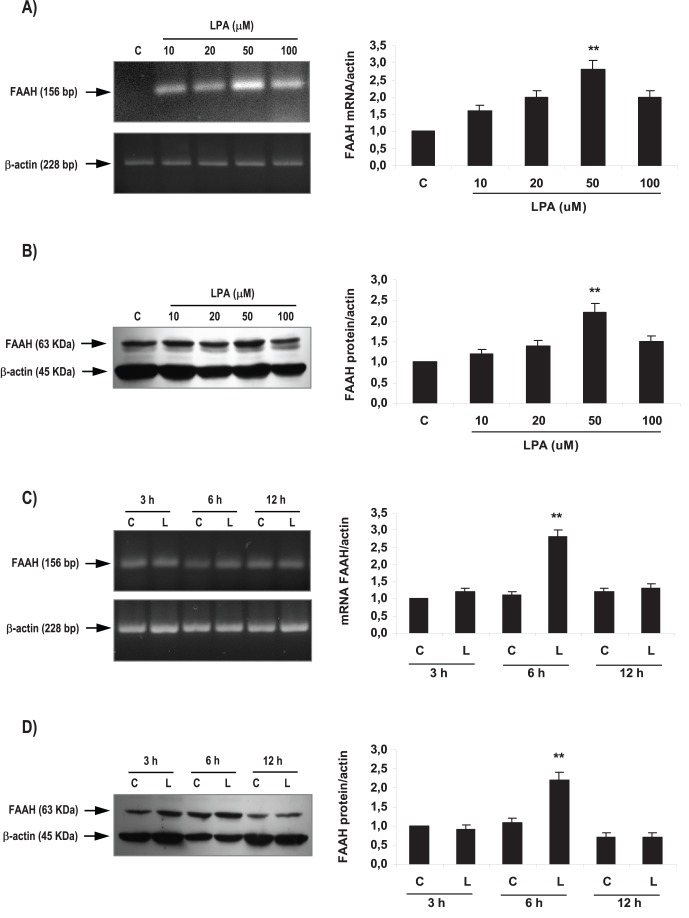
Effect of LPA on FAAH activity and expression in the rat uterus during the window of implantation. Uterine strips from rats pregnant on day 5 of gestation (implantation window) were incubated with different concentrations of LPA (10, 20, 50 and 100 µM) for 6 h and the expression of FAAH mRNA (A) and protein (B) was studied. Afterwards, uterine strips from rats pregnant on day 5 of gestation (implantation window) were incubated with LPA 50 µM for 3, 6 and 12 h and the expression of FAAH mRNA (C) and protein (D) was determined. In A, B, C and D: ** p<0,01 vs the rest. Results are shown as means ± sem. N = 4–6 for each point. C: control; L: LPA.

### Ethics Statement

The experimental procedures reported here were approved by the Animal Care Committee of the Centro de Estudios Farmacológicos y Botánicos (CEFYBO - CONICET) and by the Institutional Committee for the Care and Use of Laboratory Animals, Permit Number: 2550/2010 (CICUAL, Comité Institucional para el Cuidado y Uso de Animales de Laboratorio) from the Facultad de Medicina (Universidad de Buenos Aires), and were carried out in accordance with the Guide for Care and Use of Laboratory Animals (NIH). All animals were provided by the animal facility of the Facultad de Odontología (Universidad de Buenos Aires).

**Figure 3:**
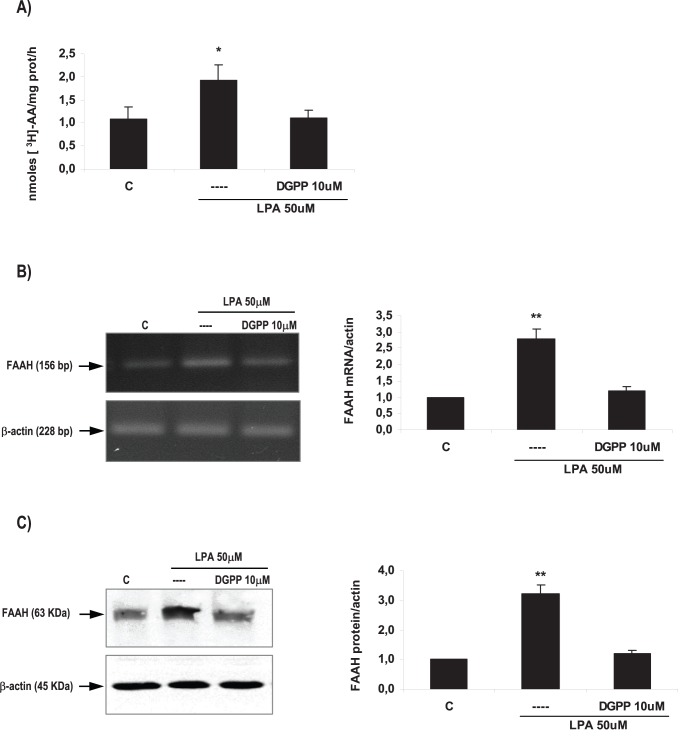
LPA3 mediated LPA effect on FAAH activity and expression in the rat uterus during the window of implantation. Uterine strips from rats pregnant on day 5 of gestation (implantation window) were incubated with LPA 50 µM for 6 h in the presence of DGPP 10 µM (a selective LPA3 antagonist) and the activity (A) and the expression of FAAH mRNA (B) and protein (C) were determined. In A: * p<0,05 vs the rest; In B and C: ** p<0,01 vs the rest. Results are shown as means ± sem. N = 4–6 for each point. C: control.

### Animals

Female rats of the Wistar strain were housed in group cages under controlled conditions of light (12 h light, 12 h dark) and temperature (23–25°C). Animals received food and water *ad libitum*. Where mentioned, animals were sacrificed in a carbon dioxide chamber and all efforts were made to minimize suffering.

Virgin female rats were mated with fertile males of the same strain. The morning the spermatozoa were observed in the vaginal fluid was defined as day 1 of pregnancy. Under the conditions of our animal facilities, spontaneous term labor occurs on day 22 of gestation.

**Table 3:** Effect of DGPP 10 µM on FAAH activity and expression.

	FAAH activity	FAAH expression	
	nmoles [^3^H]-AA/mg prot/h	FAAH mRNA/actin	FAAH protein/actin
**control**	1,2±0,2	1,0±0,0	1,0±0,0
**DGPP 10 µM**	1,1±0,2	1,1±0,2	1,2±0,1

Uterine strips from rats pregnant on day 5 of gestation were incubated for 6 h with DGPP 10 µM. FAAH activity was determine by radioconversion and FAAH mRNA and protein expression was analyzed by RT-PCR and Western Blot respectively.

Implantation is the process by which embryos make a close physical and physiological contact with the maternal endometrium for the establishment of pregnancy (for details see reviews [22], [23]). In rats, implantation occurs in the evening of 5 days post coitus and is preceded by embryo spacing, uterine edema and luminal closure resulting in an intimate apposition of the blastocyst with the uterine luminal epithelium. Around this precise moment, the endometrium acquires the ability to implant the developing embryo within a specific time window, termed the “receptive phase” or “window of implantation”. During this period, the endometrium undergoes pronounced structural and functional changes induced by the ovarian steroids, estrogen and progesterone, which prepare it to be receptive to invasion by the embryo. Thus, rats on days 5 of pregnancy were sacrificed at 9∶00–10∶00 in the morning (implantation window) and uterine horns were excised. Tissues were immediately cultured as described below (*in vitro* studies), frozen at −70°C (western blot), fixed in paraphormaldehyde (immunohistochemistry) or homogenized in Trizol (RT-PCR).

Also, rats pregnant on day 4 (prior to implantation) and day 6 (after implantation) of gestation were sacrificed and the uteri were processed for RT-PCR assays. On day 6 of pregnancy distinct macroscopically visible uterine swellings indicated the implantation sites and the uterine horns were separated into implantation and inter-implantation sites.

**Figure 4:**
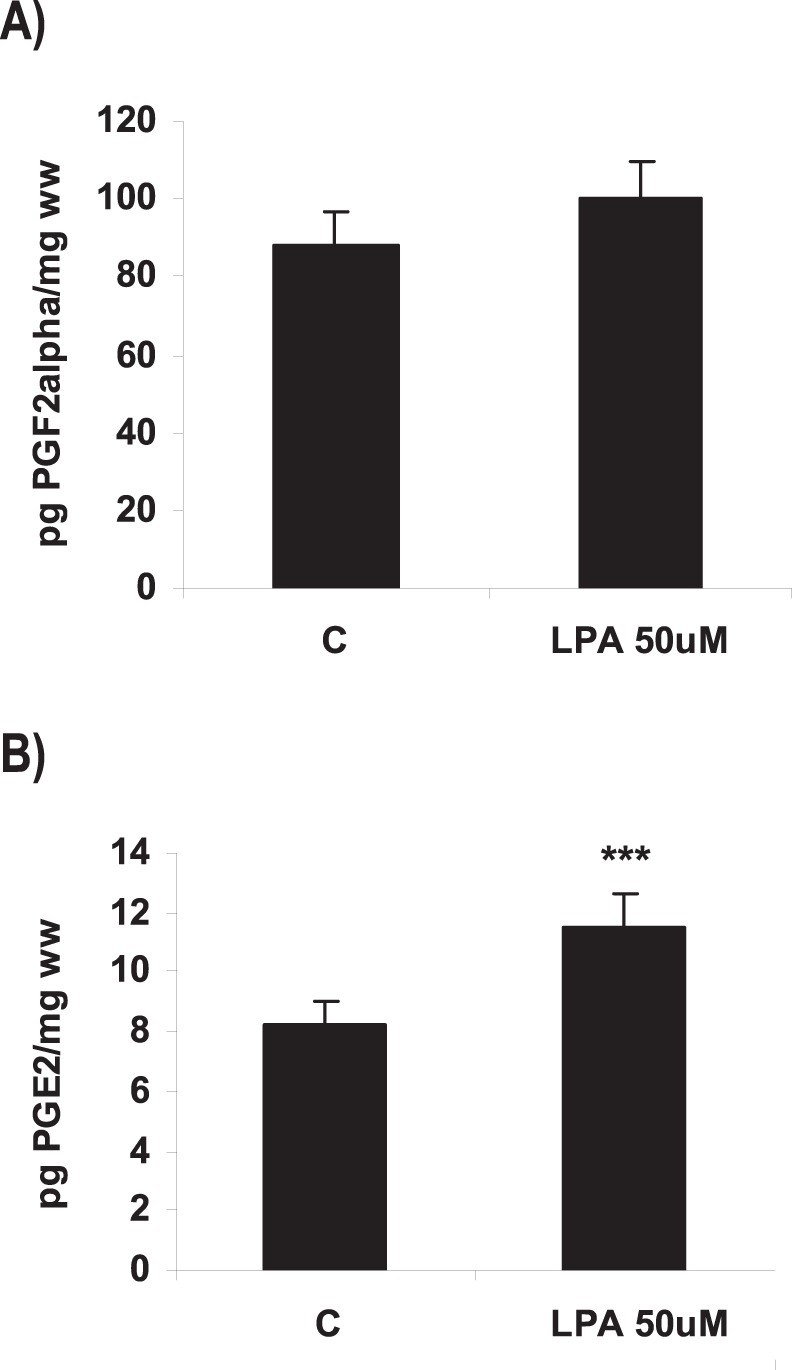
Effect of LPA on the production of prostagandins in the rat uterus during the window of implantation. Uterine strips from rats pregnant on day 5 of gestation (implantation window) were incubated with LPA 50 µM for 6 h and the production of PGF2α (A) and PGE2 (B) was determined by radioimmunoassay. In B: *** p<0.001 vs C. Results are shown as means ± sem. N = 4–6 for each point. C: control.

**Figure 5:**
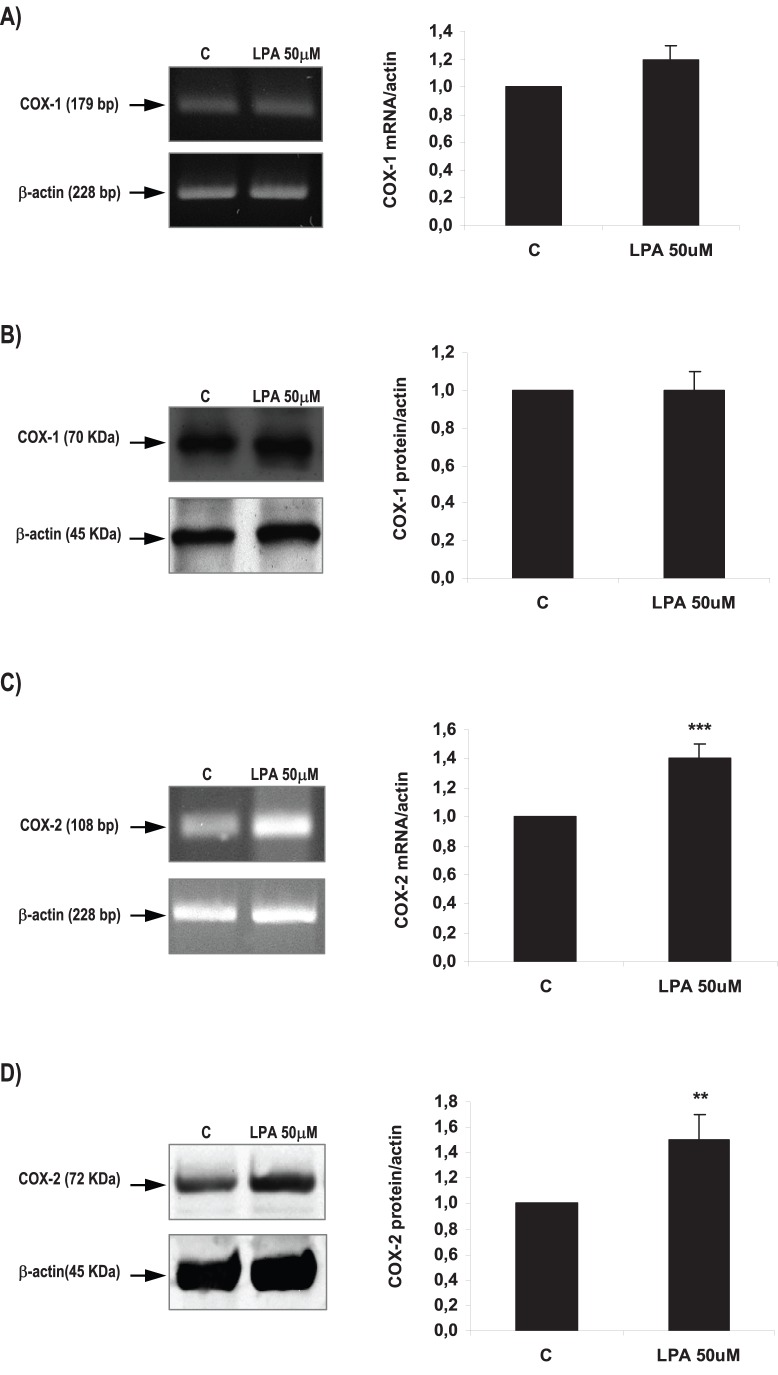
Effect of LPA on COX-1 and COX-2 expression in the rat uterus during the window of implantation. Uterine strips from rats pregnant on day 5 of gestation (implantation window) were incubated with LPA 50 µM for 6 h and the expression of COX-1 mRNA (A) and protein (B) and of COX-2 mRNA (C) and protein (D) was studied. In C: *** p<0,001 vs C; In D: ** p<0,01 vs C. Results are shown as means ± sem. N = 4–6 for each point. C: control.

### *In vitro* Studies

Uterine slices from rats pregnant on day 5 of gestation were weighted and incubated in 24 wells plates. Where mentioned, the tissue was incubated with different drugs (LPA, DGPP, indomethacin, NS-398, AM251, AM630) in Dulbecco’s Modified Eagle Medium supplemented with fetal calf serum 10%, penicillin G 20 IU/ml, streptomycin 20 µg/ml and amphotericin B 50 ng/ml (final volume: 500 µl). Cultures were maintained in 5% CO2 in air at 37°C. After the culture, tissues were immediately frozen at −70°C (western blot, FAAH activity) or homogenized in Trizol (RT-PCR). All supernatants were immediately frozen at −70°C (prostaglandins’ radioimmunoassays).

**Table 4:** Effect of LPA and COX inhibitors on the production of PGE2.

	pg PGE2/mg ww
**control**	8,3±0,4
**LPA 50 µM**	11,9±1,5^a^
**LPA 50 µM + indomethacin 1 µM**	7,5±0,7
**LPA 50 µM + NS-398 1 µM**	7,4±0,5
**indomethacin 1 µM**	9,4±0,6
**NS-398 1 µM**	6,5±1,4

a :p<0.001 vs the rest.

Uterine strips from rats pregnant on day 5 of gestation were incubated for 6 h with LPA 50 µM alone or in the presence of indomethacin 1 µM (a non selective COX-1 and COX-2 inhibitor) or NS-398 1 µM (a selective COX-2 inhibitor). In another set of experiments, the tissue was incubated for 6 h with indomethacin or NS-398 alone. The production of PGE2 was determined by radioimmunoassay.

### Immunohistochemistry

Uteri from pregnant rats on day 5 of gestation were removed and fixed overnight in paraphormaldehyde 4% in phosphate-buffered saline (PBS) 0.1 M (pH 7.4). The tissue sections were dehydrated and embedded in paraffin. The paraffin block was orientated to enable the implantation sites to be sectioned transversally. Sections of 5 µm were made by a microtome (Leica RM 2125, Wetzlar, Germany) and mounted on 2% silane-coated slides. The sections were stained with hematoxylin–eosin, and observed by light microscopy (Nikon Eclipse 200, NY, USA) to determine general tissue morphology and to identify the different cell types present. For immunohistochemistry studies, the samples were blocked of endogenous peroxidase with hydrogen peroxide 0.3% v/v in methanol for 10 min and rinsed with PBS. The slides were pre-incubated with nonimmune rabbit serum diluted in PBS (1∶100) in a humidity chamber at room temperature for 1 h. Then, the slices were incubated at 4°C overnight in a humidity chamber with a polyclonal rabbit antibody directed against LPA3 (1∶50 in PBS). Negative controls were incubated omitting the first antibody. The immunoperoxidase technique was then performed following the protocol for the RTU Vectastain kit. The antigen was revealed by diaminobenzidine. Finally, the sections were dehydrated, counterstained and mounted for observation.

**Figure 6:**
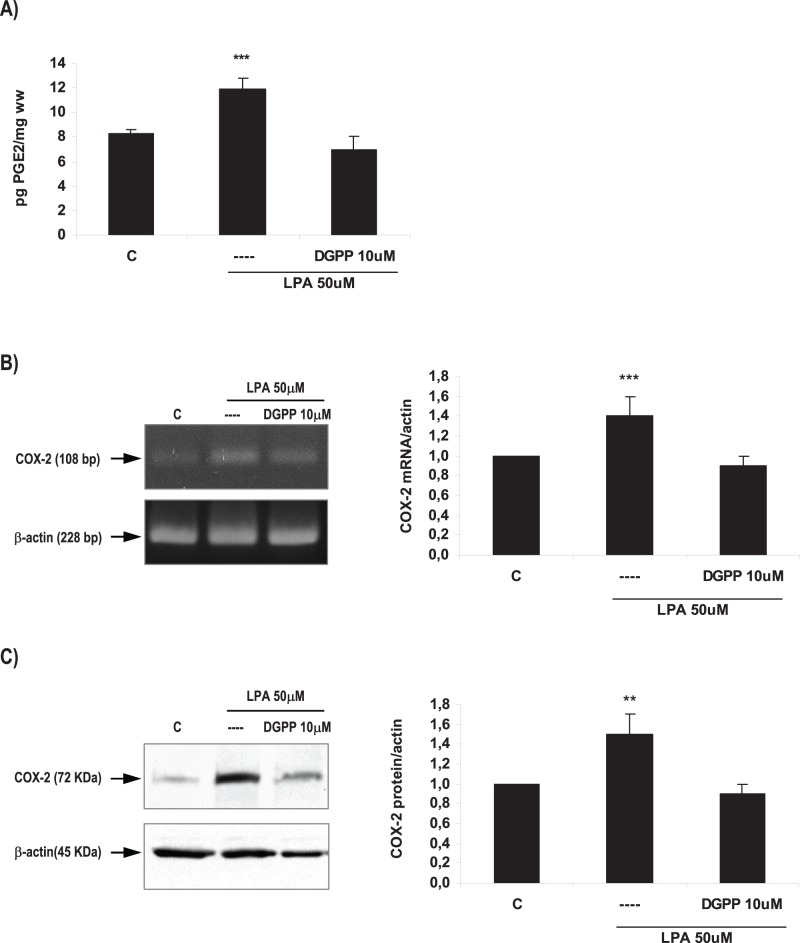
LPA3 mediated LPA effect on the production of PGE2 and on COX-2 expression in the rat uterus during the window of implantation. Uterine strips from rats pregnant on day 5 of gestation (implantation window) were incubated with LPA 50 µM for 6 h in the presence of DGPP 10 µM (a selective LPA3 antagonist) and the production of PGE2 (A) and the expression of COX-2 mRNA (B) and protein (C) were determined. In A and B: *** p<0,001 vs the rest; In C: ** p<0,01 vs the rest. Results are shown as means ± sem. N = 4–6 for each point. C: control.

### Polymerase Chain Reaction Analysis (RT-PCR)

Total RNA obtained under different conditions was isolated using Trizol reagent according to the manufacturer’s recommendations. RNA was thawed on ice, quantified spectrophometrically at 260 and 280 nm and RNA quality assessed using ethidium bromide-stained gels. RNA with a 260∶280 ratio of 1.8 and above was further treated with RNAse free DNase I to digest contaminating genomic DNA. First strand cDNA was synthesized from total RNA (3 µg) using Moloney murine leukemia virus reverse transcriptase (MMLV-RT) and random primers according to the manufacturer’s recommendations (Invitrogen, Buenos Aires, Argentina) in the presence of ribonuclease inhibitor. After first strand synthesis, polymerase chain reaction (PCR) was performed with specific intron spanning primers. PCR primers are specified in Table 1. The PCR conditions in all cases started with a denaturation step at 94°C for 5 min and followed by up to 35 cycles of denaturation, annealing and primer extension (Table 1). PCR products were resolved in 2% agarose gels and visualized by ethidium bromide staining. Photographs were taken using a digital camera Olympus C-5060 and analysed using the Image J software package (open source). Data were expressed as the relative amount of LPA3, Lyso-PLD, FAAH, COX-1, COX-2, IGFBP-1 or IL-10 versus β-actin mRNA.

### Western Blotting

Uterine slices frozen at –70°C were incubated in triple-detergent buffer (PBS pH = 7.4 with sodium azide 0.02% w/v, SDS 0.1% w/v, Nonidet P-40 1% v/v, sodium deoxycholate 0.5% v/v) containing leupeptin 10 µg/ml, aprotinin 2 µg/ml, soybean-trypsin inhibitor 100 µg/ml, EDTA 1 mM, benzamidine 1 mg/ml, DTT 10 µg/ml and caproid acid 1 mg/ml. Tissues were homogenized (Ultra Turrax, T25 basic, IKA Labortechnik), sonicated for 30 s (Ultrasonic Cell Disrupter, Microson, Heat systems Inc.) and centrifuged for 30 min at 20000 g. Protein determination was assayed by the Bradford method [24] using bovine serum albumin as standard. Equal amount of proteins (100 µg/lane) were separated in 7.5% (COX-1, COX-2 and Lyso-PLD), 10% (FAAH) or 12% (LPA3) w/v SDS-PAGE (15 mA at room temperature) and subsequently transferred to nitrocellulose membranes (30 V at 4°C for 18 h). Specific positive controls were also loaded. Non-specific binding sites of the membranes were blocked using dried non-fat milk 5% w/v in PBS pH = 7.4. Membranes were incubated with the corresponding primary antibodies followed by a goat anti-rabbit horseradish peroxidase-conjugated IgG (Table 2). Both the first and the second antibodies were diluted in PBS. Non-specifically bound antibody was removed by washing three times with PBS containing Tween-20 0.1% v/v. Immunoreactive bands were visualized and photographed using Image Quant Software (GE Healthcare, Buenos Aires, Argentina). Immunoreactive specificity was assessed by omitting the first antibody. Protein bands were identified by molecular weight markers. β-actin was used as loading control. The intensity of bands was determined using the Image J software package (open source). Results were expressed as relative optic density LPA3, Lyso-PLD, FAAH, COX-1 or COX-2/β-actin.

**Table 5:** Effect of DGPP 10 µM on the production of PGE2 and on the expression of COX-2.

	pg PGE2/mg ww	COX-2 mRNA/actin	COX-2 protein/actin
**control**	8,3±0,4	1,0±0,0	1,0±0,0
**DGPP 10 µM**	10,4±1,1	1,2±0,2	0,9±0,1

Uterine strips from rats pregnant on day 5 of gestation were incubated for 6 h with DGPP 10 µM. The production of PGE2 was determined by radioimmunoassay. COX-2 mRNA and protein expression was analyzed by RT-PCR and Western Blot respectively.

### Prostaglandins Radioimmunoassay

PGF2α and PGE2 concentrations in the culture supernatants were determined by radioimmunoassay [25] as previously described [26]. The PGF2α and PGE2 antiserum were highly specific and showed low crossreactivity. Sensitivity was 5 pg per tube and ka = 1.5×10^10^ M. Values were expressed as pg of PGF2α or PGE2/mg wet weight.

### Determination of Fatty Acid Amide Hydrolase (FAAH) Activity

FAAH (EC 3.5.1.4) activity was assayed as previously described by Paria and colleagues [27] and by our laboratory [28]. Briefly, uterine slices were weighted, homogenized (Ultra Turrax, T25 basic, IKA Labortechnik) and sonicated in buffer Tris-HCl 10 mM (pH = 7.6) and EDTA 1 mM. Homogenates (100 µg) were incubated at 37°C for 15 min in 200 µl of Tris-HCl 50 mM (pH = 8.5) containing [^3^H]-anandamida 100 µM (172.4 Ci/mmol, 100 µCi/ml) and anandamide 8 µM. The reactions were terminated by the addition of chloroform:methanol (1∶1 v/v). The aqueous phase was extracted twice with chloroform and pooled extracts were dried. Dried samples were resuspended in chloroform and seeded in a TLC plate. The hydrolyzed [^3^H]-arachidonic acid was resolved in the organic layer of a solvent system of ethyl acetate:hexane:acetic acid:distilled water (100∶50:20∶100 v/v) mixture. The plate was exposed to iodine to identify the zones corresponding to arachidonic acid. The distribution of radioactivity on the plate was counted in a scintillation counter by scraping off the corresponding spots detected in the plate. The area of each radioactive peak corresponding to arachidonic acid was calculated and expressed as a percentage of the total radioactivity of the plates. Protein concentration was determined by the method of Bradford [24]. Enzyme activity is reported as nmol [^3^H]-arachidonic acid/mg protein/h. The optimal reaction conditions were previously determined (data not shown).

**Figure 7:**
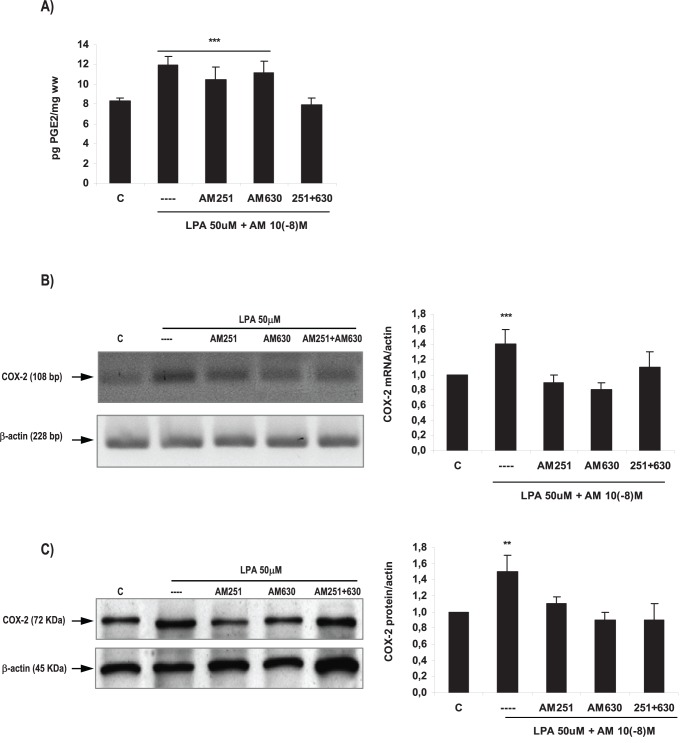
Endocannabinoids mediated LPA effect on the production of PGE2 and on COX-2 expression in the rat uterus during the window of implantation. Uterine strips from rats pregnant on day 5 of gestation (implantation window) were incubated with LPA 50 µM for 6 h in the presence of AM251 10**^−^** ^8^M (a selective CB1 antagonist) or AM630 10**^−^** ^8^M (a selective CB2 antagonist) or both. The production of PGE2 (A) and the expression of COX-2 mRNA (B) and protein (C) were determined. In A: *** p<0,001 vs C and 251+630; In B: *** p<0,001 vs the rest; In C: ** p<0,01 vs the rest. Results are shown as means ± sem. N = 4–6 for each point. C: control.

### Statistical Analysis

Statistical analysis was performed using the GraphPad Prism Software (San Diego, CA, USA). Comparisons between values of different groups were performed using one way ANOVA and t-Student. Significance was determined using Tukey’s multiple comparison tests for unequal replicates. A number of 4–6 animals were used for each treatment. All values presented in this study represent means ± S.E.M. Differences between means were considered significant when p was 0.05 or less.

**Table 6:** Effect of cannabinoid receptors’ antagonists on the production of PGE2 and on the expression of COX-2.

	pg PGE2/mg ww	COX-2 mRNA/actin	COX-2 protein/actin
**control**	8,9±0,4	1,0±0,0	1,0±0,0
**AM251 10^−8^M**	10,5±0,6	1,2±0,2	0,9±0,1
**AM630 10^−8^M**	10,3±1,8	0,8±0,2	0,9±0,1

Uterine strips from rats pregnant on day 5 of gestation were incubated for 6 h with AM251 10^−8^M (a selective CB1 antagonist) or AM630 10^−8^M (a selective CB2 antagonist). The production of PGE2 was determined by radioimmunoassay. COX-2 mRNA and protein expression was analyzed by RT-PCR and Western Blot respectively.

## Results

### Characterization of LPA3 and Lyso-PLD Expression and Localization

We observed that the rat uterus expressed both the mRNA (Figure 1A) and the protein (Figure 1B) of LPA3 receptor during the peri-implantation period (days 4 to 6 of pregnancy). LPA3 messenger was amplified and identified by RT-PCR in the uterus obtained from rats pregnant on days 4 to 6 of gestation as a single band at 221 bp (Figure 1A). The expression of this receptor was increased in the day of implantation (day 5). After invasion begins (day 6), LPA3 mRNA was higher in the inter-implantation sites compared to the implantation sites. LPA3 protein was detectable as a single band at the expected molecular mass of 40 KDa in all the cases analyzed (Figure 1B). With respect to LPA3 localization, it was detectable in the endometrium obtained from rats on day 5 of gestation (Figure 1C). LPA3 was localized to the apical membrane and subapical cytoplasm of the luminal and glandular epithelium. In immunohistochemistry studies, no staining was observed when the first antibodies were omitted (Figure 1C).

**Figure 8:**
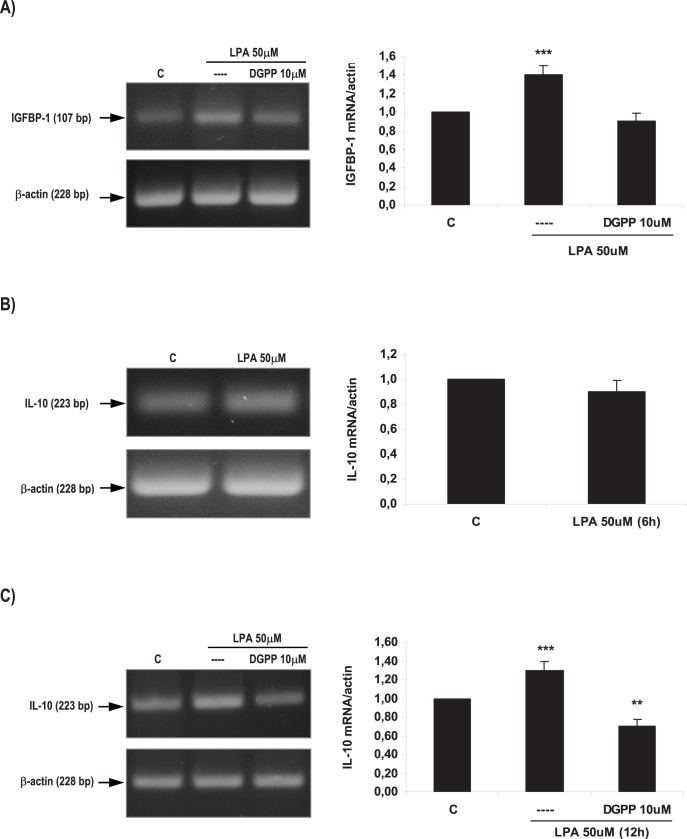
Effect of LPA on decidualization and vascularization markers in the rat uterus during the window of implantation. Uterine strips from rats pregnant on day 5 of gestation (implantation window) were incubated with LPA 50 µM for 6 and 12 h and the expression of IGFBP-1 (A) and IL-10 (B and C) was studied. In A: *** p<0,001 vs the rest; In C: *** p<0,001 vs the rest, ** p<0.01 vs C. Results are shown as means ± sem. N = 4–6 for each point. C: control.

Lyso-PLD messenger was also amplified and identified by RT-PCR in the rat uterus at the window of implantation as a single band at 247 bp (Figure 1D). Lyso-PLD protein expression was readily detectable in the studied tissue and it appeared as a band at approximately 100 KDa (Figure 1E). Two immunoreactive bands of higher molecular weight were also observed that most likely corresponded to glycosylated forms of the enzyme, as indicated in the manufacturer data sheet.

**Figure 9:**
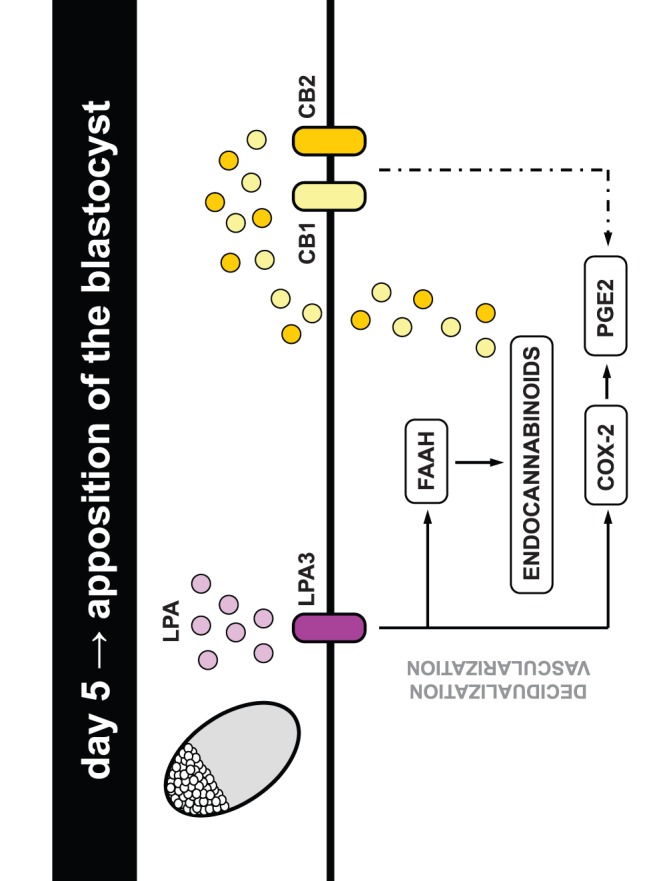
Model of interaction between LPA, prostaglandins and endocannabinoids at the implantation site.

### LPA Increased FAAH Expression and Activity through LPA3 Receptors

As previously mentioned in the introduction section, several reports indicate the participation of LPA3 and the endocannabinoid system in the process of implantation. Thus, we decided to investigate if LPA modulated the expression and the activity of FAAH at the window of implantation. It has been reported that FAAH is one of the main enzymes that degrade endocannabinoids in the uterus of pregnant mice [19].

In order to determine optimal incubation conditions, concentration-response and time-response curves were carried out using uterine tissues obtained from rats pregnant on day 5 of gestation (window of implantation). Uterine strips were incubated with different concentrations of LPA (10, 20, 50 and 100 µM) during 3, 6 and 12 h (Figure 2). The selected concentrations of LPA were based on the physiological concentration found in plasma of women in the first trimester of gestation [12]. We observed that the incubation with LPA 10, 20 and 100 µM for 6 h did not modify the expression of FAAH. However, the incubation with LPA 50 µM for 6 h augmented the expression of both FAAH mRNA (Figure 2A) and protein (Figure 2B). Thus, afterwards, day 5 uterine strips were incubated with LPA 50 µM for 3, 6 and 12 h. Only the incubation during 6 h augmented the expression of FAAH mRNA (Figure 2C) and protein (Figure 2D). In subsequent experiments we incubated the tissues with LPA 50 µM for 6 h.

After setting up the experimental conditions, we investigated if LPA modulated FAAH activity. We observed that the incubation with LPA 50 µM for 6 h also augmented the activity of FAAH in the rat uterus during the window of implantation (Figure 3A).

To study if LPA3 receptor was mediating LPA effect on FAAH expression and activity during the window of implantation, uterine strips from day 5 of gestation were pre-incubated for 30 min with DGPP 10 µM (a selective antagonist of LPA3). Afterwards, tissues were incubated for 6 h with LPA 50 µM alone or in the presence of the antagonist. Based on both binding and functional data described in the data sheet, DGPP at the selected concentration is a highly potent and selective antagonist for LPA3 receptor. We observed that the incubation with DGPP 10 µM blocked LPA effect on FAAH activity (Figure 3A), FAAH mRNA expression (Figure 3B) and FAAH protein level (Figure 3C). The incubation with DGPP 10 µM alone did not regulate FAAH activity and expression (Table 3). These results suggest that during the window of implantation, LPA stimulated FAAH activity and the expression of FAAH mRNA and protein through the activation of LPA3 receptor.

### LPA Increased COX-2 Derived PGE2 Production via LPA3 Receptors

It has been reported that exogenous administration of PGE2 and carbaprostacyclin (a stable analog of PGI2) into LPA3^−/−^ females rescues delayed implantation [2], [8] and that LPA regulates the production of PGs in different reproductive tissues [9]–[11]. Thus, we analyzed if LPA via LPA3 receptor modulated the production of PGs and the expression of COXs isoforms in the rat uterus during the window of implantation. We observed that during the window of implantation the incubation of uterine strips with LPA 50 µM for 6 h did not modify the production of PGF2α (Figure 4A), but augmented PGE2 levels (Figure 4B). While LPA 50 µM for 6 h did not regulate COX-1 mRNA (Figure 5A) and protein (Figure 5B), it increased the expression of both COX-2 mRNA (Figure 5C) and protein (Figure 5D) in the rat uterus. This result suggests that LPA increased COX-2 derived PGE2 production in the rat uterus during the window of implantation. In order to confirm that COX-2 was the isoform involved in the increase in PGE2 production under the effect of LPA, uterine strips from day 5 of gestation were pre-incubated for 30 min with indomethacin 1 µM (a non selective inhibitor of COX-1 and COX-2) or NS-398 1 µM (a selective COX-2 inhibitor). Afterwards, tissues were co-incubated for 6 h with LPA 50 µM and indomethacin or NS-398 and PGE2 production was determined. The incubation with LPA + indomethacin or LPA + NS-398 prevented the stimulatory effect of LPA on PGE2 production to the control levels (Table 4), suggesting that LPA increased the production of PGE2 through the activation of COX-2 isoform. Neither indomethacin 1 µM nor NS-398 1 µM incubated alone presented any effect on PGE2 production (Table 4).

As previously described in this section, to study if LPA3 receptor was mediating LPA effect on PGE2 production and COX-2 expression during the window of implantation, uterine strips from rats pregnant on day 5 of gestation were pre-incubated for 30 min with DGPP 10 µM (a selective antagonist of LPA3). Afterwards, tissues were incubated for 6 h with LPA 50 µM alone or in the presence of the antagonist. PGE2 production and COX-2 mRNA and protein expression were determined. The incubation with the selective antagonist of LPA3 receptor blocked the effect of LPA on PGE2 production (Figure 6A) and on COX-2 mRNA (Figure 6B) and protein (Figure 6C) expression. The incubation with DGPP 10 µM alone did not modify the basal production of PGE2 and the expression of COX-2 mRNA and protein (Table 5). Thus, LPA stimulates COX-2 derived PGE2 production through the activation of LPA3 receptor in the rat uterus during the window of implantation.

### Endocannabinoids Participated in the Effect of LPA on COX-2 Derived PGE2 Production

We have recently published that AEA stimulates the production of PGE2 and PGF2α in the receptive rat uterus through the activation of CB2 receptors [21]. In the present work, we observed that LPA modulated FAAH activity and expression, the production of PGE2 and the expression of COX-2 mRNA and protein. It has been described that FAAH is the endocannabinoids’ main degrading enzyme. Thus, we decided to investigate if endocannabinoids were mediating the effect of LPA on COX-2 derived PGE2 production. To test this hypothesis, uterine strips obtained from rats pregnant on day 5 of gestation were pre-incubated for 30 min with AM251 10**^−^** ^8^M (a selective CB1 antagonist) or AM630 10**^−^** ^8^M (a selective CB2 antagonist). Then, tissues were co-incubated for 6 h with LPA 50 µM alone or with AM251 10**^−^** ^8^M or AM630 10**^−^** ^8^M. PGE2 production and COX-2 expression were determined. Based on both binding and functional data, AM251 and AM630 at the selected concentration are highly potent and selective antagonists for CB1 and CB2 receptors respectively [29], [30]. We observed that the co-incubation of LPA with both AM251 and AM630 prevented the stimulatory effect of LPA on PGE2 production (Figure 7A). When we studied the expression of COX-2, we observed that the incubation with AM251 or with AM630 or with both antagonists together, reversed the stimulatory effect of LPA on COX-2 mRNA (Figure 7B) and protein (Figure 7C). These results suggest that endocannabinoids participated in the effect of LPA on the production of PGE2 and the expression of COX-2 during the window of implantation. The incubation with AM251 or AM630 alone did not modify the production of PGE2 or the expression of COX-2 (Table 6).

### LPA Increased the Expression of Decidualization and Vascularization Markers

To test if LPA, via LPA3, promotes the process of implantation regulating decidualization and vascularization at the feto-maternal interfase, we evaluated the expression of two molecular markers of decidualization (IGFBP-1, insulin growth factor binding protein 1) and vascularization (IL-10) under the effect of LPA (for details see reviews [31], [32]). Thus, uterine strips obtained from rats pregnant on day 5 of gestation were pre-incubated for 30 min with DGPP 10 µM. Afterwards, tissues were incubated for 6 h and 12 h with LPA 50 µM alone or in the presence of DGPP 10 µM. The expression of IGFBP-1 and IL-10 was determined by RT-PCR. We observed that LPA 50 µM for 6 h increased the expression of IGFBP-1 mRNA in the rat uterus (Figure 8A). Although, LPA 50 µM for 6 h did not modify the expression of IL-10 mRNA (Figure 8B), the incubation of the rat uterus with LPA 50 µM for 12 h significantly augmented the expression of IL-10 mRNA (Figure 8C). The incubation with DGPP, the selective antagonist of LPA3 receptor, blocked the effect elicited by LPA on the expression of both molecular markers (Figure 8A and 8C). The incubation with LPA and DGPP decreased the expression of IL-10 beyond the control value (Figure 8C).

## Discussion

In the present study, we showed for the first time that LPA3, a G protein-coupled receptor essential for implantation, and Lyso-PLD, the main enzyme described so far responsible for LPA production, are expressed in the rat uterus during the window of implantation. Also, LPA3 was found to be localized in the uterine endometrium, which is in close contact with the invasive trophoblast. The fact that LPA3 is differentially regulated during the peri-implantation period suggests that the expression of this receptor depends on the presence of the blastocyst and its state of activation.

In pigs, LPA3 expression was highest near implantation, localized to the luminal and glandular epithelium and elevated by estrogen in the endometrium [9]. In this species, as well as in rodents and humans, estrogen plays a critical role during the time of embryo implantation. In this sense, mice LPA3 was localized to the luminal endometrial epithelium [2] and both progesterone and estrogen cooperatively regulate its expression [33], thereby contributing to the receptivity of uteri during early pregnancy.

As previously mentioned, Lyso-PLD is a key enzyme for the production of LPA [34]–[36]. The production of LPA by Lyso-PLD activity in serum is considerably increased during human pregnancy [12], indicating the physiological role of LPA production by Lyso-PLD in blood circulation on maintenance of pregnancy [12], [13]. Using immunohistochemical stain of Lyso-PLD of placenta from three trimesters of pregnancy, Iwasawa and colleagues [14] suggested that trophoblasts might produce Lyso-PLD, and so LPA, since the first trimester. Here, we observed that the rat uterus expresses Lyso-PLD enzyme during the window of implantation, suggesting that the rat uterus is capable of producing LPA during this stage of pregnancy. Seo and colleagues [9] reported that the porcine endometrium produces LPA, as LPA with various fatty acyl groups is present in the uterine lumen in both the estrous cycle and pregnancy. Apart from the platelet-aggregating and smooth muscle-contracting effects of LPA [37], [38], much attention has been directed to its regulatory effects on the integrity and function of vascular endothelial barrier [39]–[41].It is possible that the presence of Lyso-PLD in the rat uterus during the window of implantation might be related to the vascular remodeling at the maternal-fetal interfase, which is essential for the successful maintenance of pregnancy and, thus, to achieve normal gestation.

We observed that the incubation of uterine strips obtained from rats pregnant on day 5 of gestation (window of implantation) with LPA 50 µM increased FAAH activity and expression and also augmented the production of COX-2 derived PGE2 production. The effective concentration of LPA used in this work is within the physiological range reported in different corporal fluids. With increasing length of gestation, the elevated Lyso-PLD activity in pregnant women was found to produce increasing proportions of LPA that reaches a serum concentration of ∼50 µM in the first trimester of gestation [12]. Additionally, LPA was found to be present in human follicular fluid [42] and uterine flushings of domestic mammals [9], [11] at micromolar concentrations. Also, we described that the maximum effect was observed after 6 h of culture. We hypothesized that a downregulation of LPA3 receptors may exist after 6 h causing a decrease in the response elicited by LPA on FAAH expression.

Here we show that the incubation with LPA did not modify PGF2α production while it significantly increased the levels of PGE2. Some authors found an increase of uterine PGE2 and PGF2α is observed on day 5 of pregnancy, allowing the decidualization process to take place [43]. However, during postimplantation, PGE2 returns to the original preimplantation levels, but PGF2α decreases [43]. Thus, whereas PGE2 contributes to the process of decidualization, implantation and recognition of pregnancy, an increase in PGF2α over certain values can terminate pregnancy [44]. A high level of PGF2α is known to induce inhibition of implantation, alteration of embryo development and induction of luteal regression [45]. In fact, the group of Callegari [46] suggested that the abortificient role of elevated levels of PGF2α is due, in large part, to inhibition of genes involved in the normal turnover of the extracellular matrix necessary for decidual formation.

We decided to test if an LPA3 antagonist reversed LPA action, as here, as well as in pigs and mice [2], [9], we observed that LPA3 is differentially expressed in the rat endometrial epithelium, which is in close contact with the embryo tissues. Luminal endometrium localization in mice differentiates LPA3 from the other six LPA receptors and may underlie its essential role in embryo implantation [47]. Also, deletion of LPA1 and LPA2 in mice revealed roles for these receptors in neural development, craniofacial formation, neuropathic pain and altered cellular signaling, but without obvious effects on female reproduction [48]–[51]. However, embryo implantation studies identified clear phenotypic changes in LPA3-deficient dames: delayed implantation and altered positioning/crowding of embryos [2]. Furthermore, patients displaying recurrent implantation failure expressed reduced levels of LPA3 and COX-2 in the endometrium compared with normal patients [52]. In the present work we observed that the incubation with a selective LPA3 antagonist completely blocked the stimulatory effect of LPA on FAAH, COX-2 and PGE2. Taken together, these results suggest that LPA signaling in female reproduction is mediated by LPA3.

As previously mentioned, we observed that LPA increased COX-2 derived PGE2 production. Prostaglandins play a fundamental role at the site of implantation regulating decidualization and vascularization, two main processes during embryo invasion. COX-2 is restricted to implantation sites in most species studied and COX-2^−/−^ mice have defective implantation and decidualization [3], [5]. PGE2 and PGI2 increase vascular permeability and decidualization at the implantation sites [6], [7] and exogenous administration of PGE2 and PGI2 into LPA3^−/−^ females rescues delayed implantation but did not rescue defects in embryo spacing [2], [8]. Other authors also showed that LPA stimulates the expression of COX-2 mRNA in the porcine endometrium [9] and increases the synthesis of PGE2 in the ovine trophectoderm and in the bovine endometrium [10], [11]. These data identify LPA3 receptor-mediated signaling as a new influence on implantation and further indicate linkage between LPA signaling and PGs biosynthesis. In this sense, the group of Seo and colleagues [9] suggested that LPA produced in the uterine endometrium may play a critical role in uterine endometrial function and conceptus development through LPA3-mediated COX-2 expression during implantation and establishment of pregnancy in pigs.

The fact that LPA increased FAAH activity and expression and also COX-2 expression suggests that LPA might regulate endocannabinoid levels in the rat uterus during the window of implantation. Genetic evidence demonstrates that FAAH is the major degrading enzyme for AEA [19]. Besides FAAH, COX-2, monoacylglycerol lipase and to some extent COX-1 participate in metabolizing 2-AG in the pregnant uterus [19]. Here we observed that endocannabinoids mediated LPA effect on COX-2 derived PGE2 production, thus suggesting that these three groups of lipid molecules interact during the window of implantation. Previously, we have reported that the blastocyst intrinsic program might operate in conjunction with ovarian hormones regulating nitric oxide levels via cannabinoid receptors in a specific manner during implantation [53].

The importance of LPA-LPA3 signaling during implantation is reinforced by the results showing that LPA through LPA3 receptor seemed to increase normal decidualization and vascularization. Previous reports supported by the findings obtained in the present work reinforced the notion that LPA3 has a critical role in regulating implantation. It is possible that certain abnormal pregnancies, such as ectopic pregnancy and placenta previa, are due to attenuated LPA3 signaling. Besides, the endometrium from women with endometriosis shows decreased expression of LPA3 in the midsecretory and late secretory phases, suggesting that decreased expression of this receptor may indicate impaired endometrial receptivity in these patients [54].

Recurrent embryo implantation failure is a disorder with potentially devastating physiological and psychological manifestations for those affected. The results presented in our study strongly suggest that lipid related molecules specially LPA and its receptor LPA3, and endocannabinoids and its receptors CB1 and CB2 (Figure 9), could be used as markers for the receptivity of the uterus during early pregnancy and seem to be promising therapeutic targets for overcoming female infertility caused by the lack of uterine receptivity.
